# Peri-operative kidney injury and long-term chronic kidney disease following orthotopic heart transplantation in children

**DOI:** 10.1007/s00467-014-2878-4

**Published:** 2014-08-14

**Authors:** Aparna Hoskote, Michael Burch

**Affiliations:** 1 Cardiac Intensive Care and ECMO, Institute of Child Health, Great Ormond Street Hospital for Children NHS Foundation Trust, Great Ormond Street, London, WC1N 3JH UK; 2 Cardiothoracic Unit, Great Ormond Street Hospital, Great Ormond Street Hospital for Children NHS Foundation Trust, Great Ormond Street, London, UK

**Keywords:** Renal failure, Chronic kidney disease, Renal insufficiency, Calcineurin inhibitor, Complications, Creatinine clearance, Bridging to transplant

## Abstract

Significant advances in cardiac intensive care including extracorporeal life support have enabled children with complex congenital heart disease and end-stage heart failure to be supported while awaiting transplantation. With an increasing number of survivors after heart transplantation in children, the complications from long-term immunosuppression, including renal insufficiency, are becoming more apparent. Severe renal dysfunction after heart transplant is defined by a serum creatinine level >2.5 mg/dL (221 μmol/L), and/or need for dialysis or renal transplant. The degree of renal dysfunction is variable and is progressive over time. About 3–10 % of heart transplant recipients will go on to develop severe renal dysfunction within the first 10 years post-transplantation. Multiple risk factors for chronic kidney disease post-transplant have been identified, which include pre-transplant worsening renal function, recipient demographics and morbidity, peri-transplant haemodynamics and long-term exposure to calcineurin inhibitors. Renal insufficiency increases the risk of post-transplant morbidity and mortality. Hence, screening for renal dysfunction pre-, peri- and post-transplantation is important. Early and timely detection of renal insufficiency may help minimize renal insults, and allow prompt implementation of renoprotective strategies. Close monitoring and pre-emptive management of renal dysfunction is an integral aspect of peri-transplant and subsequent post-transplant long-term care.

## Introduction

Significant advances in heart transplantation medicine have improved survival outcome in children with end-stage heart disease [1–3]. In addition, extracorporeal mechanical circulatory support (MCS) offers these children with single- or multi-organ failure an opportunity to wait for a suitable organ to become available. With the introduction of highly effective immunosuppressive treatment, the 1-year and long-term graft survival rates have markedly improved. However, with improved survival, several detrimental effects of long-term immunosuppression, in particular renal dysfunction and consequent chronic renal insufficiency (CRI), have emerged, all of which increase the risk of post-transplant morbidity and mortality [4–8]. Renal insufficiency is an important cause of morbidity after paediatric transplantation, with the majority of patients experiencing at least mild renal dysfunction [9–12]. The specific focus of this review is acute kidney injury (AKI) following heart transplantation in children; chronic kidney disease (CKD) requiring dialysis and future renal transplant are also briefly discussed.

## Defining the spectrum of renal dysfunction

### Acute kidney injury

Acute kidney injury may be defined using the AKI diagnostic criteria and staging system as proposed by the Acute Kidney Injury Network (AKIN), which is based on acute alterations in serum creatinine and/or urine output [13]. AKI immediately post-transplant may be defined as a ≥50 % increase in the baseline pre-operative creatinine value in the first 1–2 post-operative weeks and/or the need for early post-operative dialysis. In one adult study, post-transplant acute renal failure was defined on the basis of a serum creatinine level of ≥0.3 mg/dL (26.4 μmol/L) or a ≥ 50 % rise in serum creatinine from the pre-operative value within the first 7 days after heart transplant (HT) and/or the need for early post-operative dialysis [14].

### Chronic renal insufficiency

In the ISHLT (The International Society for Heart and Lung Transplantation) Registry, CRI is defined as a serum creatinine level of >2.5 mg/dL (221 μmol/L) and has been used to describe post-transplant renal morbidity [2]. CKD is defined as a glomerular filtration rate (GFR) of <60 mL/min/1.73 m^2^ for at least 3 months and/or signs of kidney damage for ≥3 months [15]. CKD, further defined in stages (1–5) depending on severity, may be used to categorize degrees of renal impairment after transplant.

### Severity

The degree of severity of renal dysfunction may be variable after HT. In the ISHLT Registry, severe renal dysfunction is defined as a serum creatinine level of >2.5 mg/dL (221 μmol/L) and the need for dialysis or renal transplant [2, 3]. End-stage renal disease (ESRD) has been defined by an ongoing need for dialysis and/or having had renal transplant.

## Measuring renal dysfunction: challenges

The GFR remains the most widely accepted indicator of renal function, and its determination by nuclear medicine scintigraphy is the gold standard measurement method. However, as formal GFR measurement is not practical for routine monitoring, the most commonly used method is the estimated GFR (eGFR) by the Schwartz method [16]. It is important to note that there are significant limitations in accurately assessing renal insufficiency with this method. In particular, identifying mild to moderate renal dysfunction may be challenging. Studies in the transplant population comparing the two methods have shown that eGFR significantly underestimates the burden of renal insufficiency [10, 12, 17]. In a longitudinal study of children post-HT, eGFR overestimated the measured GFR (by nuclear medicine scintigraphy) by 33 ± 26 mL/kg/1.73 m^2^ [10]. English et al. found that the GFR estimated by creatinine clearance consistently overestimated the GFR and that the latter was >2 standard deviations below the mean normal value in 38 % of their study cohort [17]. Creatinine is a function of muscle mass, and height is a surrogate for lean body mass. Transplant recipients may have normal serum creatinine values in the face of incipient renal dysfunction because creatinine generation may be low due to reduced muscle mass and poor nutritional state from end-stage heart failure, possibly explaining the discrepancy in eGFR and measured GFR in the above-mentioned studies. Furthermore, it can be challenging to measure renal function in infants [18] and children aged <2 years, and in this age group GFR may be better expressed as the percentage of normal GFR for age and gender (GFR % > 75, indicating normal renal function) [9].

## Prevalence and progression of renal dysfunction

Renal dysfunction is one of the most common medical morbidities post-solid organ transplantation [3, 19–21]. Although the cause may be multifactorial in origin, it is mainly related to the side-effects of long-term immunosuppression [4, 7, 17, 19, 22]. The prevalence of CKD following non-renal paediatric transplantation is often underestimated—and is often diagnosed late. Based on published studies, the prevalence of renal dysfunction after lung transplant (LT) is higher than after HT, or even after heart–lung transplant (H–LT), and is perhaps related to the higher level of immunosuppression in LT recipients [2, 3, 11]

### Acute kidney injury

In the immediate post-transplant period, AKI may result following renal hypoperfusion and a low cardiac output state in association with long graft ischaemic times [23], isolated right heart failure [24] and acute graft failure [25]. AKI may also be seen in the context of acute drug-induced nephrotoxicity related to the use of calcineurin inhibitors (CNI) and/or aminoglycoside antibiotics, or it may be seen as part of multi-organ failure due to sepsis. In a national UNOS Registry study, Tang et al. found that 4.8 % of paediatric HT recipients developed AKI requiring dialysis [8]. AKI usually tends to improve with renal replacement therapy (RRT) and very rarely may be the cause of death in the first month after HT [3]. However, a paediatric HT study [8] and an adult study [14] have shown that AKI in the first week post-HT associated with a longer duration of mechanical support increased hospital stay and increased early mortality.

AKI is not limited to the immediate peri-operative period and can occur at any time in the post-transplant period; it is usually associated with changes in immunosuppressive drug levels, graft function, rejection episodes, dehydration or infection.

### Chronic renal insufficiency

Multiple single-centre studies and ISHLT Registry data have demonstrated a variable progression of renal dysfunction over time, with most studies showing an early decline in the first 6–12 post-transplantation months followed by a gradual decline over subsequent years [9, 17, 26–28]. On longitudinal follow-up of renal function using measured GFR, Bharat et al. found at least mild renal impairment in 16 and 66 % of patients at 1 and 5 years after HT, respectively [10]. In another single-centre study with a median follow-up of 5 years post-HT, there was progressive increase in renal insufficiency from 17 to 21 % and then to 29 % at 1, 3 and 5 years, respectively [27]. In a 10-year U.S. study of paediatric HT recipients (age <18 years) from 1990 to 1999 who survived for >1 year, 3 % developed ESRD during the mean follow-up period of 7 years (range 1–14 years). The 5-year actuarial risk for ESRD was 0.9 % and that for CRI was 4 %, both increasing to 4.3 and 11.8 % at 10 years, respectively [5]. In a more recent study from the PHTS registry, 1.4 % developed ESRD over a follow-up period of 4 years, and freedom from late renal dysfunction was 71 and 57 % at 5 and 10 years, respectively [6]. In the ISHLT Fifteenth Official Paediatric Registry Report 2012, 10 % of HT recipients required RRT in the form of dialysis or renal transplant by 15 years post-transplant [3].

In contrast, very few studies have shown an improvement or even long-term stability of renal function over time [29, 30]. Phan et al. reported an improvement in the post-operative period, presumably as a result of better cardiac output and improved renal perfusion [30]. In a single-centre study of thoracic transplant recipients, among whom HT recipients predominated, Pradhan et al. found a decline in the mean percentage of normal eGFR in the first 6 months after transplantation, following which the level remained relatively stable for nearly 9 years. However, the GFR % (percent normal for age and gender), showed a significant decline over time within all age brackets, with the maximum decline occurring in the first 2 years despite adjustment for improvement in nutritional state. In addition, the percentage of patients with a GFR % of >75 dropped from 78 % at the time of transplant to 45 % at 1 year, 29 % at 2 years and only 14 % at 5 years after transplantation [9].

The variability in the above studies reflects the variation in indicators used for measuring renal dysfunction, the shortcomings in estimating equations based on serum creatinine and the differences in immunosuppression protocols in different centres. These studies are tabulated in Table 1.

**Table 1:** Outline of studies related to post-heart transplant renal function in children

Study (first author)/year/location	Type	Patients	Renal function test	Follow-up period	Immunosuppression used	Renal functional outcome	Identified risk factors for renal dysfunction
Hornung [26]/2001/Newcastle, UK	Single-centre retrospective cohort study	HT 1985–1998 *n* = 54	GFR by Schwartz formula at ages 1, 2, 4 and 8 years	Median 5 years	Cyclosporine	Progressive decrease in mean GFR	•Early cyclosporine exposure during first 2 months
Pradhan [9]/2002/Children’s Hospital of Philadelphia, USA	Single-centre retrospective cohort study	HT, LT, H–LT 1988–1998, *n* = 46	GFR and GFR percent normal for age	9 years	Tacrolimus, cyclosporine, azathioprine	Significant decline in the mean % of normal estimated GFR over time in all age groups	•Younger age at HT •Higher tacrolimus levels in the first 6 months post-HT
English [17]/2002/Children’s Hospital of Pittsburgh, and Shands Hospital, Gainesville, USA	Two-centre retrospective cohort study	HT 1982–1998 *n* = 123	eGFR by Schwartz formula. mGFR, 1 month, 6 months, 1 year and then annually	7 years	Cyclosporine, tacrolimus	Steady decline, drop in creatinine clearance over time	No difference between cyclosporine and tacrolimus
Phan [30]/2003/Hospital for Sick Children, Toronto, Canada	Single-centre retrospective cohort study	HT 1994–1999 *n* = 41	eGFR by Schwartz formula, pre-HT and yearly post-HT	Mean 33 ± 17 months; 2/41 patients followed for at least 24 months	Cyclosporine, tacrolimus, methylprednisolone, MMF	Increased GFR in the first year, which remained stable. Acute renal dysfunction episodes were common	No attempts made to identify risk factors as only 3 patients had decreased GFR at follow-up.
Lee [5]/2007/ Virginia, USA	Registry study	HT SRTR 1990–1999 *n* = 2032	CRI serum creatinine ≥2.5 mg/dL (221 μmol/L) to define renal dysfunction ESRD—long-term dialysis and/or kidney transplant	Mean (range) follow-up 7 (range 1–14) years	Registry dataset, no information available on CNI dosing or drug levels	3 % developed ESRD. Children with ESRD post-HT had a ninefold increased risk of death as compared to those who did not	ESRD •HOCM •African-American race •ICU stay or ECMO at transplant •Pre-HT diabetes CRI •Pre-HT dialysis •HOCM •African-American race •Previous transplant
Sachdeva [27]/2007/Arkansas Children’s Hospital, Arkansas, USA	Single-centre retrospective cohort study	HT 1991–2004 *n* = 77	eGFR by Schwartz formula. Pre-HT and at 1, 6 and 12 months post-HT and annually thereafter	Median 5 years	Cyclosporine, azathioprine, prednisone, MMF, tacrolimus	Progressive increase in CRI post-HT	•African-American race •Younger age at HT •Longer duration of listing •CNI level
Bharat [10]/2009/Hospital for Sick Children, Toronto, Canada	Single-centre retrospective cohort study	HT 1990–2004 *n* = 91	mGFR by nuclear medicine scintigraphy	10 years	Before 1997: cyclosporine + azathioprine After 1997: tacrolimus and MMF	Freedom from mild renal insufficiency was 84 and 3 % at 1 and 5 years post-HT, respectively	•Female sex •Pre-1997 era •Higher CNI dose during first 2 months post-HT
Feingold [6]/2011/Children’s Hospital of Pittsburg, Pittsburg, USA	PHTS Database Registry Study	HT 1993–2006 *n* = 812	eGFR by Schwartz formula	Median 4.1 (range 1.5–12.6) years	Registry dataset, no information available on CNI dosing or drug levels	Late renal dysfunction	•Earlier era of HT •Black race •Rejection with haemodynamic compromise in the first year post-HT •Lowest quartile of eGFR at 1-year post-HT.
Tang [8]/2011/Children’s Hospital of Michigan, Detroit, USA	UNOS Database Registry data	HT UNOS 1993–2008 *n* = 35,98 aged <18 years	Need for dialysis	15-year dataset	Registry dataset, no information available on CNI dosing or drug levels	7 % developed PRF (dialysis from listing to hospital discharge). PRF associated with early mortality (first 6 months post-HT)	•ECMO •Ventilation •Inotrope requirement •Congenital heart disease as listing diagnosis.

CNI, Calcineurin inhibitor; CRI, chronic renal insufficiency; ECMO, extracorporeal membrane oxygenation; eGFR, estimated glomerular filtration rate; ESRD, end-stage renal disease; HT, heart transplant; H–LT, heart–lung transplant; HOCM, hypertrophic obstructive cardiomyopathy; ICU, intensive care unit; LT, lung transplant; mGFR, measured glomerular filtration rate; MMF, mycophenolate mofetil; PHTS, Paediatric Heart Transplant Study; PRF, peri-operative renal failure; SRTR, Scientific Registry of Transplant Recipients; UNOS, United Network for Organ Sharing

## Timing/pathophysiology/mechanisms of kidney injury

**Fig. 1:**
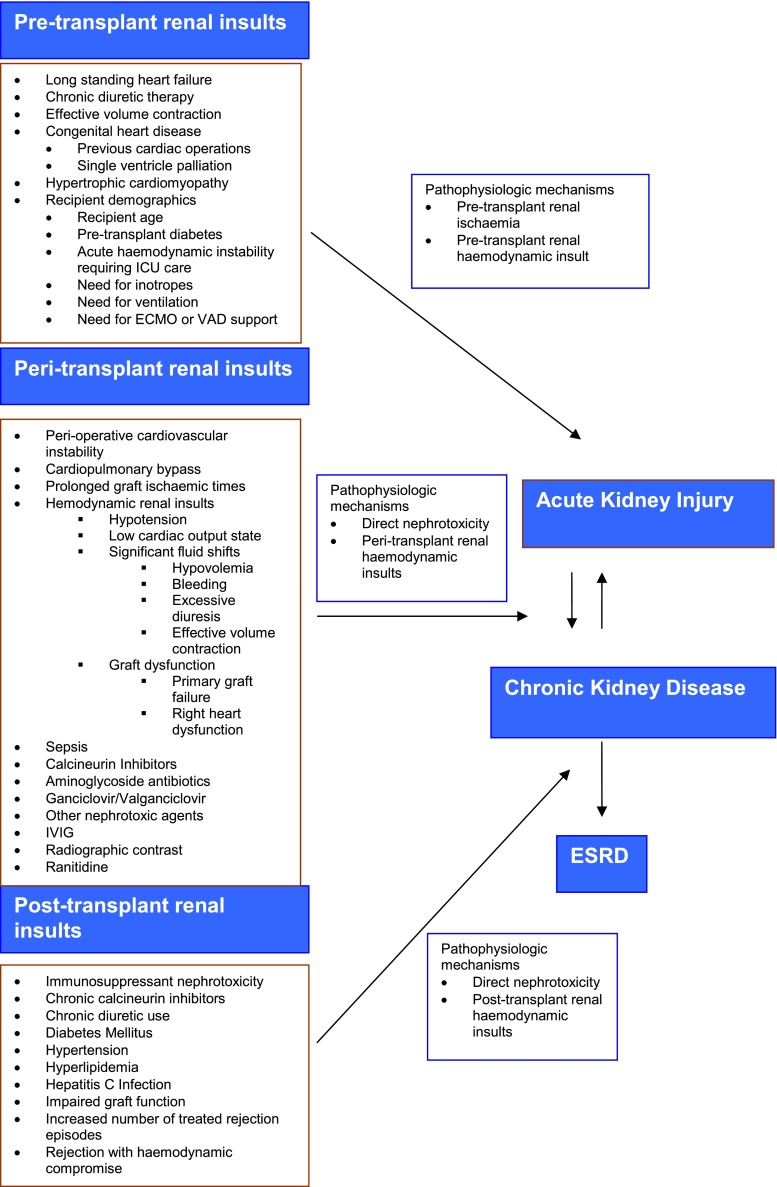
Factors contributing to kidney injury in paediatric heart transplant recipients. *ECMO* Extracorporeal membrane oxygenation, *ESRD* end-stage renal disease, *ICU* intensive care unit, *IVIG* intravenous immunoglobulin, *VAD* ventricular assist device

The factors contributing to kidney injury in paediatric HT recipients are shown in Fig. 1.

### Pre-existing (pre-transplant) renal insufficiency

Pre-existing renal insufficiency, either pre-transplant or peri-transplant, is common and may be seen in about 2.5–42 % of the study cohorts, depending on the definition used for renal dysfunction [6, 8, 9, 27, 30, 31]. Various pathophysiological mechanisms, such as renal neurohormonal activation and paediatric cardiorenal syndrome, are postulated in the context of chronic heart failure [32]. Pre-transplant renal insufficiency, however, has an important influence on post-operative renal insufficiency—the worse the degree of impaired pre-operative renal function, the higher the chances of post-operative renal dysfunction [4, 8] [30]. However, accurate assessment of renal function in the pre-transplant phase to ensure that there is adequate renal reserve is difficult because of limitations in GFR measurement. Tang et al. reported that 60 % of those on pre-transplant dialysis required it in the post-operative period and that those that needed both pre- and post-transplant dialysis had much worse longitudinal survival outcomes as compared to those who had needed it only either pre- or post-HT [8].

### Early after transplant

Varying degrees of AKI are commonly seen in the immediate peri-operative period [8, 10]. Multiple factors are responsible, including pre-transplant renal function, the transplant surgery itself with cardiopulmonary bypass, peri- and post-transplant haemodynamic state, graft function and finally nephrotoxicity from acute exposure to CNI and other nephrotoxic drugs.

#### Transplant surgery

Renal function may be affected by the transplant surgery itself, which involves cardiopulmonary bypass and aortic cross clamp against a background of a compromised general state resulting from the end-stage heart disease. In addition, there may be large volume shifts and aggressive diuresis in the immediate peri-operative period. In particular, children undergoing a high-risk transplant following single ventricle palliation, for example following Fontan surgery, who need longer duration of cardiopulmonary bypass may be vulnerable to compromised renal function [33].

#### Peri- and post-transplant haemodynamic state and graft function

In the post-operative period, particularly with HT recipients, renal perfusion is entirely dependent on the graft function. In addition, there may be significant capillary leak post-HT. Peri- and post-transplant haemodynamic state and allograft function changes, resulting in hypotension requiring inotropic support, may lead to variability in renal perfusion [4, 23, 25] and aggravate any microcirculatory abnormalities and increases in renovascular resistance caused by acute exposure to CNI agents.

Other nephrotoxic drugs in the immediate post-transplant period may further contribute to acute renal dysfunction. AKI post-HT, especially in those after failed palliation following congenital heart disease (CHD) necessitating haemodialysis, portends a poor outcome [33].

### Mechanical circulatory support in the peri-transplant period and renal function

In children with worsening heart failure, poor renal function associated with low cardiac output state is one of the indications for MCS, the rationale being that improving cardiac output will improve renal perfusion and consequently ensure good renal function. Sometimes, it may be necessary to augment MCS with RRT for a short period of time to allow for renal recovery [34]. Veno-arterial extracorporeal membrane oxygenation (ECMO) support in the context of little native cardiac function provides non-pulsatile flow to the kidneys; nevertheless, improving cardiac output and overall perfusion is most commonly associated with renal recovery. However, renal insufficiency and concomitant need for dialysis, while mechanically bridged to HT, is an important risk factor for decreased survival before and after transplant [33, 35–37].

Lately, MCS with newer ventricular assist devices (VAD), such as the Berlin Heart EXCOR device, offers paracorporeal, pneumatically driven, pulsatile assistance to the circulation [38–40]. In these critically ill children, a mild to moderate degree of renal failure is commonly seen in the pre-implantation period; not infrequently, new onset renal failure may be seen post-implantation. This is most often related to the precarious haemodynamics and poor renal perfusion pre- and peri-implantation of the device. Children with complex CHD and, in particular, those following single ventricle palliation have higher morbidity and mortality following implantation of the Berlin Heart EXCOR device [41, 42]. In a recent study by Almond et al. of children in the USA undergoing Berlin Heart EXCOR support as a bridge to transplantation, renal function (measured by GFR adjusted for age) at the time of implantation was the single strongest predictor for mortality while on the device [38].

In a single-centre study, Prodhan et al. compared the effect of MCS on renal function and found that there was a steady early improvement in eGFR in the VAD-supported group and the ECMO + VAD group which was, however, not sustained, with a subsequent decline of renal function seen over the length of support. In contrast, in the ECMO group, the improvement in eGFR continued until day 28 of support [43]. Registry-based studies have shown that the need for ECMO at the time of being listed for HT is an important risk factor for peri-operative renal failure [8] as well as for subsequent development of ESRD [5]. Tang et al. reported that the need for ECMO, ventilator and inotropic support and a primary diagnosis of CHD at listing increased the risk for peri-operative renal failure [8]. Lee et al. also reported that the need for ICU and ECMO and for pre-transplant dialysis were risk factors for ESRD and CRI, respectively [5]. These factors reflect the degree of clinical severity and highlight the level of dependency on critical care interventions, which are crucial for successful transplant outcome.

### Chronic renal dysfunction

#### Late after transplant

Chronic renal dysfunction in paediatric HT recipients is common, and is slowly progressive after an initial, early decline. The cumulative effect of total CNI exposure after transplant leads to renal tubular atrophy and interstitial fibrosis, resulting in a gradual and progressive decline in renal function [12, 44, 45].

### Immunosuppressive therapy and effects on kidney function

#### Calcineurin inhibitors

The advent of agents such as cyclosporine A and tacrolimus in post-transplant immunosuppression has revolutionized survival in transplant recipients; both of these drugs, however, are associated with direct nephrotoxicity. It is very common for transplant recipients to be on CNI agents at the 1- and 5-year follow-up [3]. Both cyclosporine A and tacrolimus cause renal ischaemia due to afferent arteriolar vasoconstriction through activation of the intra-renal renin–angiotensin system, as well as imbalances between prostaglandin E2 and thromboxane A2 effects. In an elegant review, Di Filippo et al. describe these pathophysiological mechanisms in detail [22]. Various other postulated mechanisms include an increase in endothelin-1 (a potent vasoconstrictor causing tubulointerstitial fibrosis) and a decrease in the production of nitric oxide (a vasodilator). Chronic ischaemia causes intra-renal overexpression and upregulation of transforming growth factor beta 1, which in turn promotes glomerulosclerosis and tubular interstitial fibrosis. Various gene polymorphisms may contribute to the individual variation in CNI-induced nephrotoxicity [46]. Overproduction of angiotensin II associated with the genotype of the angiotensin-converting enzyme might be associated with poor prognosis. Other indirect mechanisms for CNI nephrotoxicity have also been implicated, such as thrombotic micro-angiopathy, sodium retention, hypertension, dyslipidemia (cyclosporine A effect) and diabetes (tacrolimus effect) [7, 19, 47].

### When does toxicity occur? Is it dose dependent? Does early high dose matter? Is it related to duration of exposure? Is there a difference between CNIs? Is there any reversibility possible?

Two distinct patterns of cyclosporine A nephrotoxicity have been characterized in adult studies in the immediate post-operative and long-term follow-up periods after HT [44]. Moderate azotemia leading to AKI has been shown to be significantly more common in the first post-operative week in cyclosporine A-treated recipients than in patients treated with other agents, such as azathioprine [44], and is worse with intravenous cyclosporine A administration [14]. Although both occur with high prevalence, the early form does not appear to be a specific risk factor for the late form.

Nephrotoxicity with cyclosporine A is considered to be dose-dependent, and high early exposure leads to a progressive decline in renal function, which persists even after subsequent reduction in cyclosporine A dose [26, 48]. Although with lower dosage, the degree of renal impairment may be lower, the pathological changes in the kidney, as seen on biopsies, remain the same regardless of dose [45]. While CNIs have been implicated in renal insufficiency post-HT, some studies have failed to show a direct correlation between renal dysfunction and cyclosporine A or tacrolimus levels or doses, suggesting that these drugs may not be the sole factor responsible for evolving renal dysfunction post-transplant [49, 50]. In the ISHLT Fifteenth Official Paediatric Registry Report 2012, the type of CNI selected had no demonstrable influence on late renal function [3]. However, Pradhan et al. reported that higher tacrolimus trough levels over the first 6 months had a significant negative correlation with GFR %. Although these authors found the same correlation with cyclosporine A levels, it did not reach statistical significance [9]. Hornung et al., in an earlier study, showed that the 2-month cyclosporine A trough levels predicted lower GFR in the first year after transplant [26]. Similarly, Bharat et al. showed that the higher maximum cyclosporine A and tacrolimus dosage in the first 2 months post-transplant were both associated with declining GFR and increasing probability of an abnormal GFR over time [10].

Tacrolimus is being increasingly used as an alternative to cyclosporine A in paediatric HT patients [51]. Unlike cyclosporine A [52], it does not cause gingival hyperplasia or hirsuitism and has been shown to have superior efficacy in paediatric HT recipients [47]. In a longitudinal study of renal function in paediatric renal transplants, tacrolimus has been shown to be superior to cyclosporine A, and recipients had significantly better eGFR at 1, 2, 3 and 4 years post-transplant [53]. Initial studies showed that cyclosporine A-induced renal toxicity was higher with tacrolimus [51], but further studies have not found this association [17]. English et al. compared the nephrotoxicity of tacrolimus and cyclosporine A in 123 children followed at two HT centres over a 7-year period and found no differences in the decline of calculated creatinine clearance between the two groups [17]. However, hypertension, an important contributory factor to the development of CRI, was lower in those treated with tacrolimus [51].

CNI-induced nephrotoxicity increases with duration of exposure and has limited potential for reversibility [26, 48]. The younger transplant recipients, who are going through the normal phase of maturation of renal function, may thus be more vulnerable to the effects of decreased renal perfusion in the peri-operative period. In adults, reduction of the mean cyclosporine A dosage from 5.3 ± 0.7 to 2.3 ± 0.3 mg/kg/day between 9 and 21 months after transplantation with concurrent azathioprine therapy led to an improvement of renal function [44]. Some studies have reported that there may even be a partial improvement in renal function after elimination or dose reduction of CNIs even after years of use [54–56]. However, this result has not been consistently shown in other adult and paediatric studies, where the early loss of renal function was irreversible and did not improve despite substitution or reduction of CNI target levels [17, 48, 49].

### Other co-morbid factors contributing to CRI

Other associated medical conditions, such as hypertension [8, 17, 30, 57], diabetes [8, 58, 59] and hyperlipidaemia, may contribute to on-going renal dysfunction and add to the cumulative burden of renal insufficiency in the post-transplant phase.

## Predictive factors (pre- and post-transplant) for chronic renal dysfunction and outcome

The degree of pre-operative worsening renal dysfunction has been shown to be a significant factor for in-hospital mortality, but not for late post-transplant mortality. Rajagopal et al. showed that worsening renal function from the time of listing to time of transplantation, as defined by AKIN guidelines, was associated with early in-hospital mortality, but not with late post-transplant mortality [31]. These authors found that those with mild, moderate and severe degree of worsening renal dysfunction had adjusted odds ratios of 2.1, 2.7 and 3.6 for in-hospital mortality, respectively [31]. In adults, pre-transplant serum creatinine and renal indices have been found to have limited predictive value on late outcome after a HT operation [49, 60]. In the most recent ISHLT Registry report, pre-transplant renal support and pre-transplant creatinine were identified to be significant risk factors for 1-year mortality after HT [3].

In a recent national UNOS Registry-based study of HT recipients with peri-operative renal failure, Tang et al. found worse survival outcomes for those that needed pre- and post-transplant dialysis; significant risk factors were primary diagnosis of CHD, Hispanic ethnicity and requirement for post-transplant dialysis [8]. This peri-operative renal failure appeared to have the most impact on survival during the first 6 months after transplant [8]. However, the authors were not able to test the correlation between the duration of RRT and subsequent outcome.

Although eGFR at transplant has not been shown to be a predictive factor for later long-term renal dysfunction or outcome [6, 31], those that develop CRI post-HT have been shown to have a significantly higher (9-fold) risk of death [5]. In one study, post-transplantation renal insufficiency at 6 months predicted a ninefold increased risk for developing CKD at 5 years [27]. Similarly, another series showed that decreased eGFR at 1-year post-HT, but not at HT, predicted the onset of late renal dysfunction [6]. In a 10-year national cohort study of paediatric HT recipients with a mean follow-up of 7 years, the significant risk factors identified for CRI were pre-transplant dialysis, hypertrophic cardiomyopathy, African-American race and previous transplant; those for ESRD were hypertrophic cardiomyopathy, pre-transplant diabetes, African-American race and intensive care unit (ICU) stay or ECMO at the time of transplant (the latter is suggestive of haemodynamic instability) [5]. Increased risk of late renal dysfunction has also been reported in black people, those with rejection associated with haemodynamic compromise and those with the lowest quartile of eGFR >60 mL/min/1.73 m^2^ at 1 year post-HT [6]. In adults, post-HT hypertension has been found to be a predictor of renal insufficiency [61, 62].

Some studies have reported that higher maximum cyclosporine A and tacrolimus dosage in the first 6 months post-transplant is associated with declining GFR and an increasing probability of an abnormal GFR over time [9, 10, 26]. In contrast, the ISHLT 2012 report showed that the type of CNI selected had no demonstrable influence on late renal function [3]. Differences in CNI target levels and a higher probability of an abnormal renal function could be the influenced by the different immunosuppression regimens employed in different eras, as shown by Bharat et al. (higher in those transplanted before 1997) [10] and Feingold et al. (higher in those transplanted before 1999) [6].

Thus, in summary, among the many registry-based and single-centre studies on renal failure post-HT mentioned above, only few factors have been consistently identified to be of predictive value for identifying chronic renal dysfunction and subsequent outcome (see Table 1). In addition, there are some conflicting reports. Two papers have shown that younger age at HT is associated with late renal dysfunction [9, 27]; another, however, has shown that a transplant age of <2 months is associated with increasing GFR and decreasing probability of an abnormal GFR over time [10], whereas yet another has shown no association [5]. Similarly, gender has been shown to be a significant factor in one study, with females having a higher probability of an abnormal late GFR [10], which has not been borne out in other studies [5, 9, 27].

## Diagnosis and management of transplant recipients with renal insufficiency

### Measurement of renal function

Early diagnosis of renal impairment is important so that interventions may be instituted to prevent further ongoing damage. Regular monitoring and screening of renal function with serum creatinine and calculation of eGFR (when abnormal), formal GFR measurement, renal ultrasound scan, urine analysis for proteinuria and albumin/creatinine ratio are recommended. Most transplant centres maintain an institutional protocol for monitoring, which includes renal function. The ISHLT guidelines recommend that eGFR should be done and urinalysis obtained at least yearly in paediatric HT recipients [63]. Close surveillance and monitoring of CNI drug levels, with frequent adjustment to target therapeutic range, is vital. Regular screening for hypertension, diabetes and hyperlipidaemia is an integral part of post-transplant management [63]. In addition to regular monitoring, tests such as ambulatory blood pressure, oral glucose tolerance test and hyperlipidemia screen are recommended on an annual basis.

### Management of AKI

**Fig. 2:**
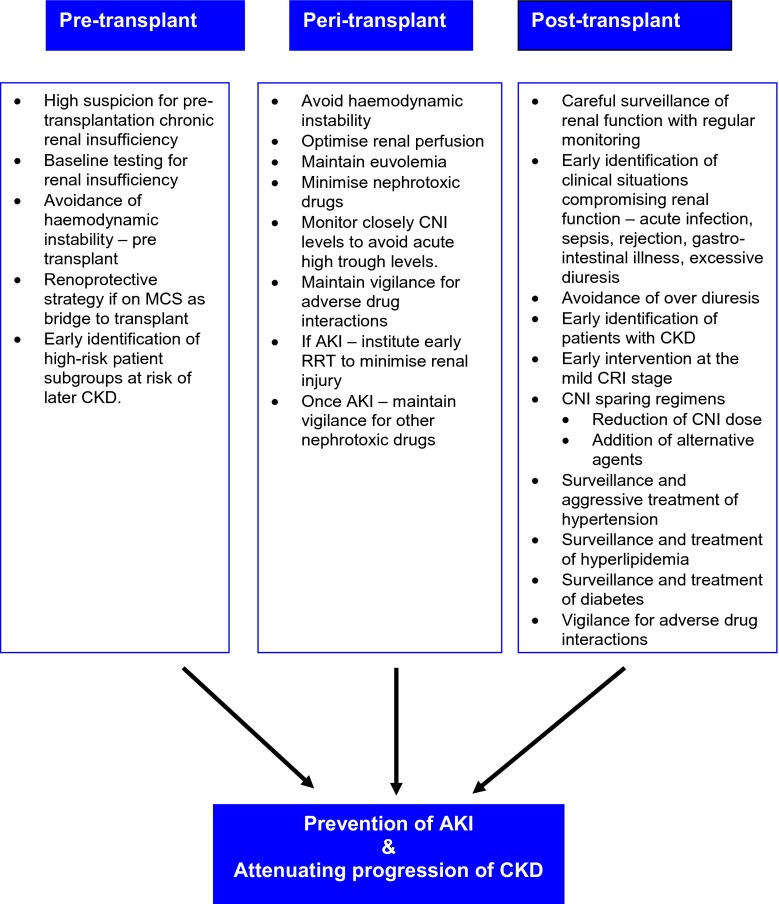
Recommendations to protect renal function and facilitate renal recovery in paediatric heart transplant recipients. *AKI* Acute kidney injury, *MCS* mechanical circulatory support, *CNI* calcineurin inhibitor, *CKD* chronic kidney disease, *CRI* chronic renal insufficiency, *RRT* renal replacement therapy

Acute kidney injury in the peri-operative period or anytime in the post-transplant period is treated with diuretics, usually loop diuretics such as furosemide, either intermittently or as an infusion with careful fluid restriction, while maintaining euvolemia and avoiding hypotension (see Fig. 2). Monitoring of central venous pressure (CVP) and targeting optimum haemodynamics to ensure renal perfusion is vital. The CVP should be maintained between 5–12 mmHg, a level that provides adequate cardiac filling pressures without causing right ventricular overload [63, 64]. In the acute phase, early continuous RRT is important to prevent fluid overload and to maintain optimum renal support. If the recipient develops anuria or oliguria or has a sharp rise in serum creatinine within 2–4 h after HT, then early continuous RRT must be instituted [63]. Similarly, if CVP remains elevated (>20 mmHg) despite pharmacologic interventions, continuous RRT must be commenced. Continuous RRT provides several advantages in critically ill post-transplant patients: (1) it can be successfully used even in haemodynamically unstable patients, including those on ECMO and fluid goals can be adjusted to suit the patient’s clinical status; (2) as it is a continuous modality, fluid restriction is not needed, which allows nutrition, blood products and medications to be provided to the patient without worsening fluid overload [65]. It is important to avoid nephrotoxic drugs—in particular antibiotics. Furthermore, a delay in the initiation of CNI therapy should be considered if there is significant pre-operative renal insufficiency or deterioration of kidney function during the first 2 post-operative days [63]. If renal failure persists beyond 4–6 weeks, then intermittent haemodialysis is an effective form of support. This modality can provide substantial clearance in a short period of time, thereby allowing the child freedom from the machine, and can facilitate rehabilitation from acute illness. However, IHD is technically challenging and requires a specialized centre with trained personnel [65].

### Management of CRI

Progressive CRI leads to many well-characterized complications, such as poor growth, anaemia, hypertension, hypercholesterolaemia, secondary hyperparathyroidism with metabolic bone disease and electrolyte abnormalities. These need to be managed with a good control of hypertension, use of lipid-lowering agents and management of metabolic bone disease (see Fig. 2). The accelerated cardiovascular disease and sodium retention associated with CKD often requires maintaining patients in a relatively volume-depleted state, which can cause further chronic ischaemic injury to the kidney [21]. Renal dysfunction is one of the factors that influences/contributes to the overall net state of immunosuppression, which in turn can predispose to an increased risk of infection [8, 66]. Interventions that have been proven to slow progression of CKD in the general population, such as management of hypertension and strict management of diabetes, must be instituted [63]. Renal dietetic review to advise on high calorie, adequate protein, low phosphate diet and potassium and oxalate dietary intake is essential.

## Facilitating renal recovery after transplantation

### Strategies to decrease CNI exposure

Strategies to improve renal function in recipients with significant renal dysfunction are directed towards reducing or eliminating exposure to nephrotoxic drugs and minimizing renal haemodynamic insults (see Fig. 2). Reducing the dose of CNI and if possible, withdrawal while substituting with another appropriate non-CNI agent—e.g. mTOR inhibitor (sirolimus/everolimus)—to maintain effective immunosuppression should be considered [56, 63, 67–71].

There have been reports that switching the adjunct drug from azathioprine to mycophenolate mofetil has allowed lower trough levels of CNIs to be maintained without significant increase in rejection [55]. More recently, the proliferation signal inhibitors sirolimus and everolimus have been used as CNI-sparing agents for immunosuppression in HT patents who have renal dysfunction without significant increase in rejection episodes [56, 67, 71, 72]. Two strategies have been used—sirolimus combined with low-dose calcineurin inhibitor and sirolimus as a replacement for calcineurin inhibitor. Both strategies have been reported to result in improved renal function, but caution has to be maintained with when sirolimus is administered without CNI as this therapy may be associated with increased rejection episodes [73]. It appears that using tacrolimus and mycophenolate mofetil provides a safer alternative to the various immunosuppressive regimens [69, 73, 74]. Basiliximab, a chimeric monoclonal antibody against CD25 (interleukin 2 receptor alfa) has potential advantages in the treatment of patients with renal impairment. Ford et al. reported that basiliximab given on Day 0 and Day 4 after transplantation in a group of ill children with pre- or post-operative renal dysfunction along with reduction or withdrawal of CNIs was well tolerated, with a low incidence of rejection [75]. Post-transplant ECMO may reduce the efficacy of basiliximab [75].

In summary, avoidance of overimmunosuppression, judicious use of steroids and CNI, minimization of doses where possible, and possibly the use of m-TOR inhibitors, are crucial in preventing and/or ameliorating renal dysfunction. In addition, careful avoidance of drugs that may alter metabolism of CNI and increase their renal toxicity is essential [76]. Furthermore, it is important to minimize or avoid exposure to other nephrotoxic drugs, such as aminoglycosides, amphotericin and ganciclovir, that are commonly used post-transplant to treat infections. Drug dosage needs to be adjusted for renal failure with careful therapeutic drug monitoring.

A word of caution needs to be emphasized: the potential risk for precipitation of rejection remains with modification of CNI agents; therefore, all these recipients must be closely followed to ensure effective immunosuppression. If significant renal insufficiency persists despite CNI reduction, the risk of precipitating rejection outweighs the benefit on renal function, and there is little evidence to support CNI-free regimens [22, 63].

## Management of ESRD

### Renal transplant

The ISHLT Fifteenth Official Paediatric Registry Report 2012 reported that 10 % of patients required RRT in the form of dialysis or renal transplant by 15 years post-heart transplant [3].

In keeping with the Kidney Disease Outcomes Quality Initiative (KDOQI) guidelines, timely preparation for RRT is essential for non-renal organ transplant recipients with declining renal function. Sequential kidney transplantation may be considered for ESRD in selected appropriate candidates [63]. Most recipients who need RRT undergo haemodialysis (HD) although chronic peritoneal dialysis (PD) may also be used; however, the risk of peritonitis remains [65]. Both forms of RRT are associated with an increased mortality rate.

### Combined or staged heart–kidney transplantation for refractory renal dysfunction

With advances in mechanical circulatory support, multi-organ transplantation with simultaneous combined heart-kidney transplant has become feasible, with reasonable outcome. Kim et al. report 2 children with end-stage heart and renal failure who were successfully bridged to combined, single-donor heart and kidney transplantation with mechanical circulatory support [77]. Simultaneous heart and kidney transplant has been reported in 0.5 % of children (UNOS database 1982–2009) where the survival was not significantly different from those undergoing heart transplant only [78]. Many of these patients (34 %) were going for a heart retransplantation. Gupta et al., in their series of recipients (UNOS database) over a 10-year period, found that the 1-year and 5-year survival after renal transplant was comparable to those who did not have a renal transplant [79].

## Prevention, intervention and education, practice and policy

Potential renoprotective strategies should be pre-emptively instituted from the peri-transplant phase (see Fig. 2). Careful attention to minimize peri-transplant renal injury, such as the avoidance of nephrotoxic drugs while awaiting transplant and optimization of peri-operative haemodynamics to prevent low cardiac output state and multiple organ failure, is important [8, 63, 64].

Certain high-risk groups, such as those with CHD and hypertrophic cardiomyopathy, may be more susceptible for renal dysfunction after HT [5, 8, 33]. Excessive diuresis leading to dehydration and effective volume contraction must be avoided while maintaining a careful watch on nephrotoxic medications.

Regular screening of renal function at 1, 3 and 12 months, and yearly thereafter, is recommended [63, 80]. Early involvement of paediatric nephrologists in the management of renal dysfunction, especially when there is renal dysfunction and a GFR of <90 mL/min/1.73 m^2^, presence of micro-albuminuria (urine albumin/creatinine 30–300 mg/g) and persistent hypertension, is recommended [63, 80]. Early detection allows the possibility to slow CKD progression or even restore function with effective management. Physicians must focus on the prevention of CKD progression, as strategies to slow progression are more effective if started early.

Modifiable risk factors to prevent the progression of CKD are control of hypertension, diabetes, hyperlipidaemia and obesity [80–82]. Patient and parent education with regard to diet, exercise, avoidance/cessation of smoking, careful attention to over-the-counter or non-prescription drugs with potential nephrotoxicity and awareness of important drug interactions is the key to successful management. Careful and perhaps preferential use of tacrolimus after transplantation is important [68, 69, 74, 75, 83]. Diligent blood pressure control with the judicious use of antihypertensive agents [81] along with low-protein diets and statin therapy may ameliorate the progression of CKD. The commonly used anti-hypertensive medications are angiotensin-converting enzyme inhibitors, calcium channel blockers [84] and angiotensin II receptor blockers but a newer agent—aliskiren, a direct renin antagonist—has been used to effectively control blood pressure resulting in slower progression of CKD [85].

## Research horizons

A newer endogenous marker of GFR, cystatin C (a low-molecular-weight protein), has been reported to be the best non-invasive estimate for the measurement of GFR in adult transplant patients [86, 87] and has been studied in paediatric HT patients along with neutrophil gelatinase-associated lipocalcin (NGAL) as a marker for AKI [88]. Cystatin C is produced at a constant rate from all nucleated cells and is more easily interpretable than creatinine as a single reference range can be used for children aged >1 year. However, these tests are expensive and as yet not readily available, and more research is needed before they can be widely accepted into clinical use. Multicentre randomized controlled trials are needed to support evidence for strategies to minimize CNI-related toxicity with alternative immunosuppressive agents without increasing the risk of rejection and compromising safety.

## Key summary points

- Peri-operative renal dysfunction is common and increases morbidity in the ICU. Severe renal dysfunction affects long-term survival in paediatric HT recipients.
- The loss of renal function is observed early in the first year after transplantation and is progressive, but continues at a slower rate with subsequent follow-up.
- The risk of CKD, ESRD increases with increasing survival.
- Appropriate monitoring and careful surveillance with longitudinal screening is recommended to detect mild renal insufficiency such that renoprotective measures can be instituted.
- Institution of CNI-sparing regimens with careful monitoring of drug levels and tailoring of immunosuppression can attenuate the progression of renal failure. However, there is an increased risk of rejection and few heart transplant patients are managed entirely CNI-free.
- A team approach with early nephrology consultation is an essential part of long-term management.

## Questions (answers are provided (following the reference list))

Question 1: The following statements regarding measurement of renal function are false EXCEPT

1. Serum creatinine is a reliable screening tool.
2. eGFR estimation by Schwartz formula is accurate in infants.
3. eGFR overestimates the degree of renal dysfunction in transplant patients.
4. Serum creatinine may be normal despite renal insufficiency in the pre-transplant period.
5. Micro-albuminuria is not used as a screening test for chronic kidney disease.

Question 2: The following statements regarding CRI post-transplant are true EXCEPT

1. Peri-operative renal dysfunction has an impact on post-transplant CRI and mortality.
2. Peri-operative AKI may get better in certain HT patients with better haemodynamics
3. Chronic calcineurin inhibitor exposure is the most common cause for progressive CRI after transplant.
4. Hypertension and hyperlipidaemia need to be controlled to minimise progression of CKD.
5. Risk of CRI decreases with increasing post-transplant survival.

Question 3: The following statements regarding risk factors for later renal dysfunction for thoracic transplant recipients are true EXCEPT

1. ISHLT Report 2012 did not find any association with type of CNI and later renal dysfunction.
2. Earlier era of transplantation has been shown to have a higher risk
3. Certain subgroups such as those with congenital heart disease are at higher risk.
4. Prolonged need for Dialysis pre- or post-transplant has been reported to be a significant risk factor for mortality after HT.
5. The number of rejection episodes in recipient has no influence on long-term CRI if appropriately managed.

Question 4: The following statements regarding CNI are true EXCEPT

1. CNI cause direct nephrotoxicity
2. CNI nephrotoxicity is dose dependent, but the effect modified by individual genetic susceptibility
3. CNI nephrotoxicity decreases with duration of exposure
4. CNI toxicity has limited potential for reversibility
5. Early high exposure leads to progressive decline in renal function

Question 5: Strategies to facilitate renal recovery include the following EXCEPT

1. Minimization of CNI dosage
2. Aggressive management of hyperlipidaemia
3. Aggressive management of hypertension
4. Vigilance for adverse drug interactions
5. Aggressive diuresis and chronic effective volume contraction
